# TNF-alpha Is Required for the Attraction of Mesenchymal Precursors to White Adipose Tissue in Ob/ob Mice

**DOI:** 10.1371/journal.pone.0004444

**Published:** 2009-02-13

**Authors:** Beatriz G. Gálvez, Nuria San Martín, Carlos Rodríguez

**Affiliations:** Projech Science to Technology, Madrid, Spain; University of Camerino, Italy

## Abstract

Most adult tissues harbour a stem cell subpopulation (Mesenchymal Precursors or MPs) that represent a small proportion of the total cell number and have the potential to differentiate into several cell types within the mesenchymal lineage. In adipose tissue, adipocytes account for two-thirds of the total cell number. The remaining cells include blood and endothelial cells, along with adipocyte precursors (adipose MPs). Obesity is defined as an excess of body fat that frequently results in a significant impairment of health. The ob/ob mice bear a mutation in the ob gene that causes a deficiency in the hormone leptin and hence obesity. Here, we present evidence that ob/ob mice have a dramatic decrease in the resident MP pool of several tissues, including squeletal muscle, heart, lung and adipose tissue. Moreover, we show that that there is a migration of MP cells from distant organs, as well as homing of these cells to the adipose tissue mass of the ob/ob mice. We call this process adipotaxis. Once in the adipose tissue, migrant MPs undergoe adipose differentiation, giving rise to new differentiated adipocytes within the adipose mass. Finally, we provide evidence that adipotaxis is largely explained by the production of high levels of Tumor Necrosis Factor-alpha (TNF-α) within the ob/ob adipose tissue. The therapeutic implications for human obesity as well as for regenerative medicine are further discussed in this paper.

## Introduction

Recent advances have implicated the adipocyte in many physiologic and pathologic processes, such as obesity, diabetes, cardiovascular disease and muscular disorders [1]. An active role for the adipocyte in energy metabolism was demonstrated with the discovery of leptin and its role in the pathogenesis of obesity [2]. The development of established cell lines from primary adipocyte precursors has greatly facilitated the study of the molecular details of adipocyte differentiation [3], [4]. Adipose tissue is also a major secretory and endocrine active organ producing a variety of bioactive proteins that may regulate energy metabolism and insulin sensitivity as well as the behaviour of the different surrounded cell types. Multipotent stem cells possess a great capacity for differentiation into the different tissues as well as tissue regeneration, but the limiting step is always that suficient numbers of reconstituting cells reach the damaged area. The success of regenerative medicine will depend on identify the mechanisms or molecules implicated in the attraction and localization of these precursors. Also the control of obesity and diabetes has proven to be difficult as long as the exact mechanism for generation and maintenance of the disease remains unknown.

## Results

**Figure 1:**
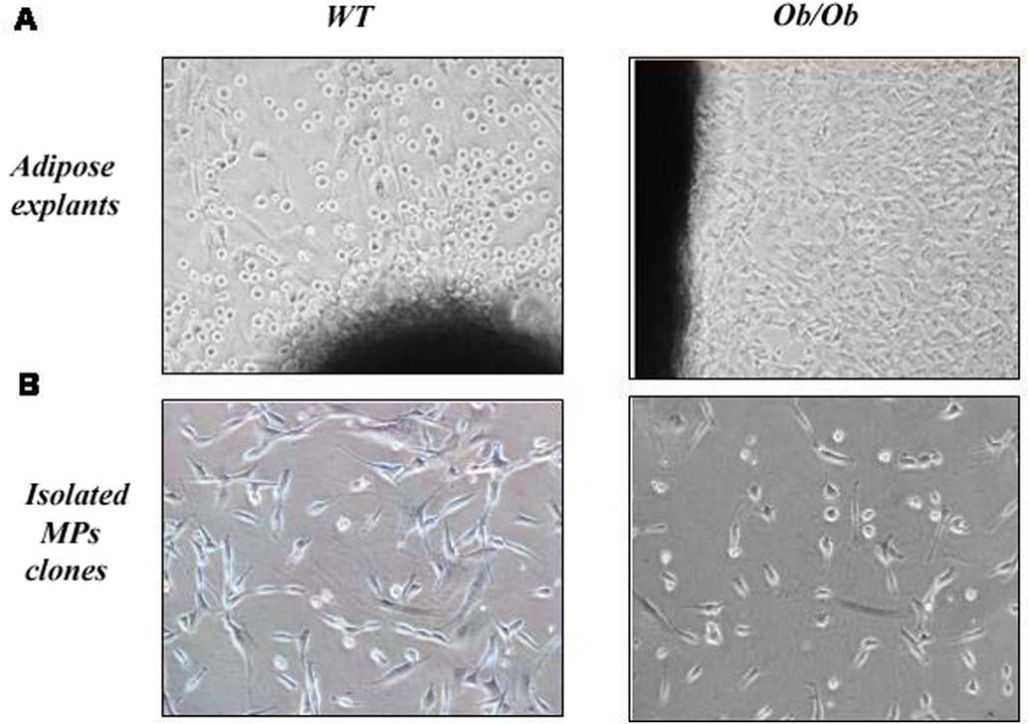
Isolation of Mesenchymal Precursors. A. MPs from adipose tissue explants in C57 wt or ob/ob mice 7 days after the surgical extraction. B. Isolated MP clones growing at subconfluence in DMEM+10%.

In order to assess the MP status in obesity, wild type C57 (wt) or ob/ob mice (leptin ko) explants (from muscle, heart, lung and adipose mass), containing organ specific MPs were taken after surgery. Three different isolation protocols (see Methods) were used for tissue processing. The explant technique was finally selected as the one with highest efficiency (see Table 1). Figure 1 shows the morphology of the MPs isolated from adipose tissue explants of both wt and ob mice. As shown in Table 1, while MP clones were easily isolated from wt explants (the average output from wt adipose or muscle tissues was more than five different clones, independently of the isolation method), the explants from ob/ob mice did yield any MP clone. This was observed in ob/ob adipose tissue explants as well as in other tissue explants (skeletal muscle, lung). Interestingly, muscles and lungs from ob/ob mice were also macroscopically smaller than the wild type.

**Table 1:** Number of MP clones isolated from the different tissues of C57 or ob/ob mice using three different techniques.

N° clones/explant	C57				OB			
	ADIPOSE	MUSCLE	HEART	LUNG	ADIPOSE	MUSCLE	HEART	LUNG
ENZYME	0	0	0	3+/−1	0	0	0	0
MECAN	12+/−3	7+/−2	0	5+/−1	0	0	0	0
EXPLANT (*)	17+/−4	11+/−3	5+/−2	9+/−3	1+/−1	0	0	2+/−1

The number of clones are statiscally significant (*p<0.05).

We also studied the proliferation properties of MPs. Wt and ob/ob mice derived MPs were cultured in the presence of DMEM+10% FBS. As shown, MPs isolated from wt mice have a slightly faster growing rate (See Figure S1). These cells were analyzed by flow cytometry and were Sca-1+, CD31+, CD34+ CD44+, CD45− and had a multipontential differentiation profile (data not shown) [5].

**Figure 2:**
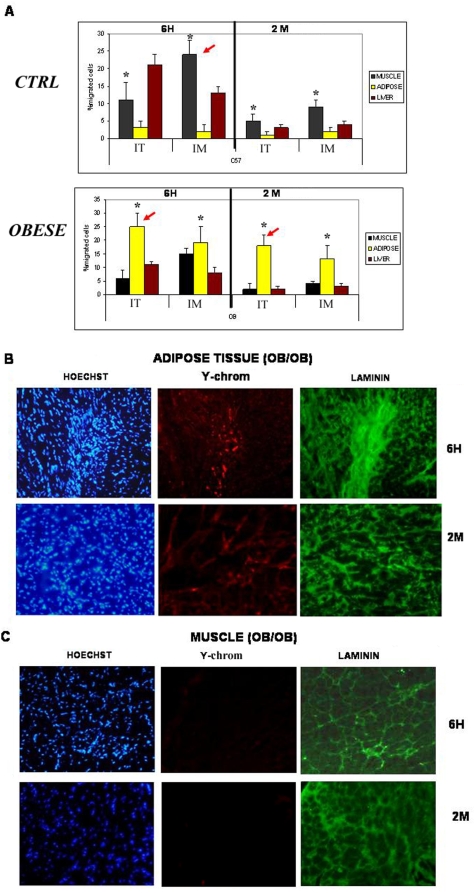
Migration and engraftment of MPs into adipose tissue. A. Results for epsilon chromosome RT-PCR, 6 h or 2 months after i.v. or i..m. male MP injection into female C57 or female ob/ob mice (*p<0.04). Note the recruitment of MPs to adipose tissue in ob/ob mice (see red arrows) (+p<0.05). B & C. Detection of intravenously injected male MPs by immunohistology in the adipose and muscle tissue of ob/ob mice 6 h or 2 months after the injection. Red fluorescence stains the injected cells, while the green color represents laminin staining (adipocytes). Hoescht dye (blue) stains all nuclei.

We then explored the likelihood that the absence of MPs in ob/ob mice could be explained by the general migration of mesenchymal stem cells from distant organs (muscle, lungs, etc.) to the adipose tissue, followed by their differentiation into mature adipocytes. This alone would explain the absence of MPs in organ tissues, including the adipose tissue. In addition, it would imply that MPs in the adipose tissue will irreversibly lose their pluripotential phenotype after undergoing adipose differentiation. To investigate this hypothesis, we injected MPs from wt males into the tail vein of wt or ob/ob females. As shown in Figure 2a, in the control mice, a variable number of injected cells could be detected in different tissues after 6 h. However, in ob/ob mice, injected MPs were almost exclusively concentrated in the adipose abdominal mass (see arrows, Figure 2a and Figure 2b). Cells were also injected intra-muscularly (im). 6 hours after im injection (Figure 2a, left panel), most of the cells remained within the muscle area in both groups of mice but, again, after such short period, we could already visualize some MPs only into the adipose mass of ob/ob mice (Figure 2c). This MPs recruitment in the adipose mass did not take place in wt mice (Figure S2). We then did a follow-up analysis of those mice two months after the injections. As shown in figure 2a (right panel), no male cells were detected in the adipose mass of C57 female mice. In the female ob/ob mice, however, male cells were still abundantly present in the adipose mass two months after both intra-venous (iv) and im injection (Figure 2a and 2b). Images of muscles and adipose tissue from injected control mice are shown in Figure S2. Moreover, these cells, independently of the tissue of origin (fat, muscle or lung) had undergone differentiation into mature adipocytes (positive for laminin, Figure 2b), thus contributing to the total adipose mass. As an additional measure of adipocyte differentiation, we assessed the expression levels of the differentiation gene marker PPAR-gamma. In agreement with the histochemistry results, positive male cells expressed the PPAR-gamma gene 15 days after the injection (Figure S3).

Injected male MPs were isolated from the adipose mass of ob/ob mice after two months and their differentiation to mature adipocytes was assessed by Red-Oil staining (see Figure S4).

To confirm directly the migration of MPs to adipose mass, we performed a transplantation assay. In this assay, we transplanted adipose mass from female ob/ob mice into male SCID mice. Three weeks after transplantation, mice were sacrificed and tissues collected. As shown in Figure S5, male cells (Y-chrom) migrate specifically into the transplanted adipose mass and could be detected by immunofluorescence. Moreover, migrated male cells were positive for red oil staining, indicating a partial or total differentiation into adipocytes (See Figure S5). We, therefore, conclude that MPs have the ability to migrate towards the adipose mass independently of their localization.

**Figure 3:**
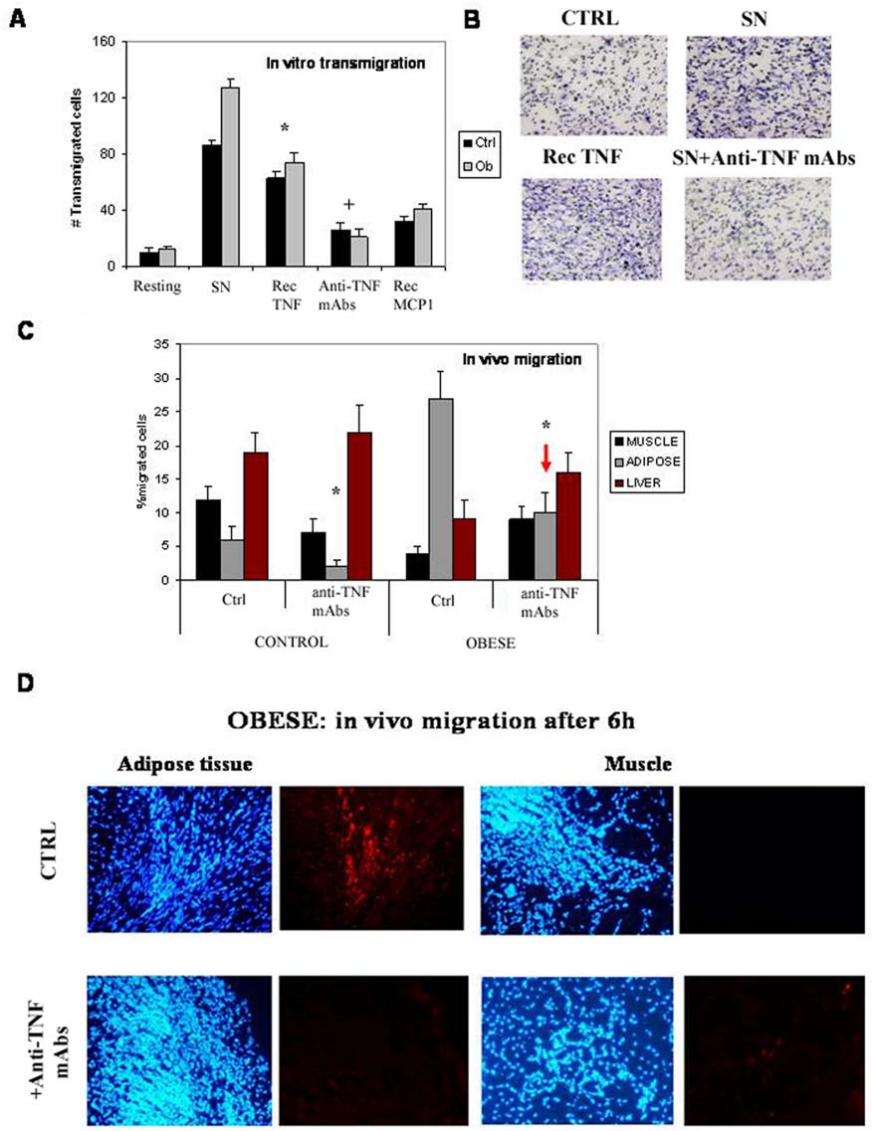
Analysis of the migration properties of MPs. A. In vitro transmigration of MPs after 6 h in the presence or absence of different conditions: mock, SN (600 µl of adypocytes supernatants), recombinant murine TNFα (Rec TNF, 50 ng/ml) (*p<0.03), anti-TNF mAbs (600 µl of adypocytes supernatants in the presence of 10 µg/ml antibodies) (+p<0.05), and MCP1 (20 ng/ml). B. Images taken from the Transwells filters after 6 h under the different conditions. C. In vivo migration into the different tissues of MP population after 6 h of C57 or ob/ob iv injection, in the presence of anti-TNF-alpha mAbs treatment (*p<0.05). Note the reduction of MPs in the adipose tissue of ob/ob mice (red arrow). D. Images taken from the adipose tissue after 6 h. Note the disappearance of injected MPs around the tissue.

In order to understand the migration mechanism of these cells, we studied their properties *in vitro*. In a transwell assay, MPs were attracted by the media conditioned by adipocyte or macrophage cultures. After analyzing different cytokines, MCP-1 and TNF-α were the best at inducing the migration of MPs *in vitro*. These cytokines are abundantly present in the adipose tissue of ob/ob mice *in vivo* and have been implicated in inflammation and angiogenesis [6], [7] (See also Figure S6). Further evidence for the role of, at least, TNF-α was found by blocking its activity with inhibitory antibodies. Indeed, anti- TNF-α antibodies strongly reduced the number of MPs that migrated across the filter (Figure 3a and 3b). These findings were confirmed *in vivo*, as MPs do not migrate to the adipose abdominal area after systemically inhibiting TNF activity with anti-TNF-α antibodies (Figure 3c and 3d). The results imply, that TNF-α is, at least, a necessary requirement for adipotaxis.

## Discussion

Current evidence suggests that, in the absence of tissue damage, systemically administered MPs seed to the bone marrow only at low levels, with large numbers of MP lodging in the pulmonary vascular bed [8], [9]. Our results show that in the ob/ob background, however, systemically injected multi-organ derived MP preferentially migrate to the adipose tissue. We hypothesize that the obese adipose tissue (at least in ob/ob mice) could behave as a surrogate injured tissue. In fact, inflammation is a hallmark of acute and chronic tissue injury, and inflammatory changes in adipose tissue are also notorious in the context of obesity [10]. In fact, several cytokines, like TNF-alpha, are produced during the inflammatory processes and some of them have already been implicated in the migration of stem cells [11], [12]

Indeed, obese adipose tissue is characterized by increased infiltration of macrophages, suggesting that they might represent an important source of inflammatory cytokines in this condition. In addition, other cellular sources like adipocytes, reticuloendothelial cells present in adipose tissue or adipocyte precursors may also contribute to these inflammatory changes [13].

Taken together, our results strongly suggest that white adipose tissue in ob/ob mice mimic an injured focus [14], promoting migration of injected MP cells from any distant localization, homing in the adipose tissue mass and, importantly, differentiating into adipocytes that are morphologically indistinguishable from resident adipose cells. This report also raise several, as yet, unanswered questions regarding the extent to which adipotaxis (and therefore MP systemic depletion) is present in other animal models of obesity (including human obesity) as well as whether it explains by itself our observation of the absence of resident MPs in several solid tissues of ob/ob mice. We have already made the observation that isolation of MPs from acute obese human patients is less effective than from control patients (data not shown).

In recent years, MPs have been increasingly given a role in tissue repair and regeneration. In different models of tissue damage, MPs improve the recovery of injured tissues, at least, through two proven mechanisms: by releasing soluble factors with anti-inflammatory activity and by promoting and potentiating local regenerative processes [15]. It is thus possible that, by sequestering MPs in the adipose tissue, obese patients could have tissue or organ repair impediments and that treatment with anti-adipotaxis agents (like anti-TNF antibodies) could prove a valuable therapeutic approach to improve repair mechanisms in organs like muscle, lung, skin, kidney, etc. Indeed, obese patients (specially those with diabetes) have impaired wound healing regardless of the degree of glucose control and insulin therapy. Moreover, in ob/ob mice, systemic anti-TNFalpha treatment restores impaired skin repair [16]. Whether our findings represent a general phenomenon of impairment of tissue repair associated with obesity remain to be proven in different situations involving tissue damage and regeneration in obese patients (ictus, myocardial infarction, chronic ulcers, etc.). In summary, we believe that the findings in this report could be of paramount importance in the search for new treatments for human obesity and its associated ailings, as well as to deal with tissue damage and repair in obese patients.

## Methods

### Mice

C57BL/6 and ob/ob mice were obtained from Charles and River, Co and maintained and used in accordance with the National Institutes of Health Animal Care and Use Committee.

### Cell isolation and growing conditions

Three isolation methods were used. In brief (see references for detailed protocols):

- *Enzymatic digestion*: Digestion of the different tissues was performed using a collagenase-dispase mixture for 30 min at 37°C, followed by centrifugation and collection of the supernatants. Cells were replated on DMEM+10% FBS (fetal bovine serum) and clones grown and selected by morphology [17].
- *Mechanical disgregation*: small pieces of tissues were disgregated using forceps and scrapels and then filtered with medium. Collected cells were plated onto plates. [18].
- *Explant technique*: Freshly collected small pieces of tissue were placed on gelatin-coated plates. Rounded cells coming out from the explant were harvested, cloned and grown to obtain MP [19]. Those clones were grown on plates covered with gelatin with DMEM+10% FBS+glut+pen/step.

### Injections

5×10^5^ MP (mixture of lung, muscle and adipose origin MP) were injected into the tail vein or into the muscle fibers with a 0.03 µm needle. Pieces of tissues of muscle, heart, liver and adipose mass were collected after 6 h or 2 months, RNA was extracted by Trizol reagent, and RT-PCR against the mouse epsilon chromosome was performed (Fw:5′GATGGTGCCTCATGGAATCT; Rw:5′AAATATGCCAAGAAGGAGAGCC). Data are represented as a percentage of migrated cells (Y-chromosome detected) to the different organs relative to the input value.

Pieces of the same tissues were kept into OCT and frozen to perform immunohistology analysis. *In situ* hybridization to mouse chromosomes from the injected MP was performed as described [20].

In the case of the treated mice, anti-TNF-α mAb (mouse anti-TNF monoclonal antibody; Endogen, MA) was administered weekly by intraperitoneal injection at 10 mg/kg, while the animals in the control group received weekly injections of saline [21].

### Statistical analysis

P values were calculated using Student's t-test. Error bars in graphs represent the standard desviation.

### Trasnwell analysis

Adipocytes and their supernatants in the presence or not of cytokines (10–50 ng/ml) or the corresponding antibodies (10 µg/ml mAb) were plated on a p24 well-plate for 2 days. At time 0 h, 8-µm transwell filters (Corning) were coated with 1% gelatin and placed onto the plate. 10^4^ MP were then plated in DMEM containing 2% serum on the upper side of transwell chamber. After 6 hours of transmigration, migrated cells on the lower side of the filter were fixed in 4% paraformaldehyde, stained with toloudine blue, and counted using an inverted microscope (five random fields of the lower face of the transwell membrane at 20× magnification). The results show migrated cells as a percentage of the total number of input cells.

## Supporting Information

Figure S1 Proliferation rate of MPs clones derived from ctrl or obese mice (p<0.05). ATP bioluminescence (RLUs) were measured in all MPs clones after 3 days of plating. (Vialight plus kit, Lonza, ME, USA)
(0.15 MB TIF)

Figure S2 Detection of intravenously injected male MPs by immunohistology in the adipose and muscle tissue of wt mice, 6 h or 2 months after the injection. Red fluorescence stains the injected cells (Y-chromose-positive), while the green colour represents laminin staining. Hoescht dye (blue) stains all nuclei.
(2.33 MB TIF)

Figure S3 Results for epsilon chromosome (grey) and PPAR-gamma (yellow) RT-PCR, 6 h or 2 months after i.v. male MPs injection into female ob/ob mice (*p<0.05). Expression analysis was performed for the differentiation marker gene PPAR_gamma. Human glyceraldehyde-3-phosphate dehydrogenase (GAPDH) was chosen as the endogenous control. Sequences of primers for PPAR_gamma: PPAR_ Fw TCAAACACATCACCCCCCTG PPAR_ Rw TGGCAGCCCTGAAAGATGC
(0.19 MB TIF)

Figure S4 Injected male MPs were extracted from the adipose mass after two months inside the mice. Y-chromosome positive cells were isolated and their adipocyte differentiation ability was tested by Red Oil staining.
(0.27 MB TIF)

Figure S5 All surgeries were performed under pentobarbital sodium anaesthesia (50 mg/kg) administered intraperitoneally. For transplantation the female adipose mass pads into male SCID mice, small bilateral dorsal incisions were made, the skin and fascia were loosened using blunt tissue forceps, and the transplants were placed under the skin, which was closed with wound clips. The peritoneum and abdominal muscles were sutured, and the skin was closed with wound clips. After three weeks, fat pads were removed, with care taken to preserve the blood vessels supplying. Transplants were processed by immunocytochemistry to detect Y-chromosome positive cells. Fat pads were also processed for the isolation of the Y-chromosome positive cells and their differentiation properties analyzed by Red Oil staining.
(1.12 MB TIF)

Figure S6 Cytokines were measured by Elisa detection kit (eBioscience). Cytokines expression is shown as ng/ml. Values are significative (*p<0.05).
(0.17 MB TIF)
