# Supplementary figures and images for: TNF-alpha Is Required for the Attraction of Mesenchymal Precursors to White Adipose Tissue in Ob/ob Mice
Source: PLoS One. 2009 Feb 13;4(2):e4444. doi: 10.1371/journal.pone.0004444 (PMC2635963; doi:10.1371/journal.pone.0004444)

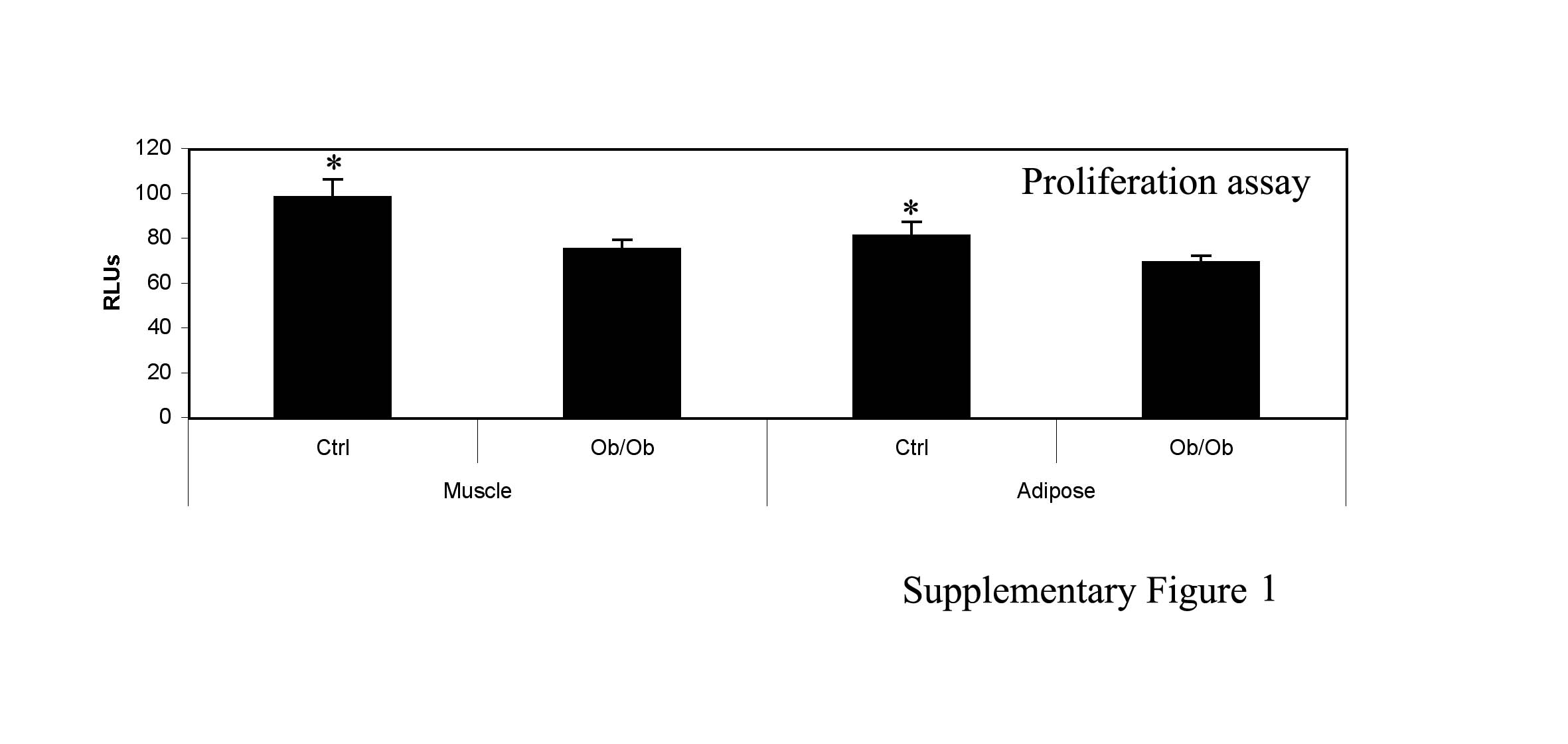

Supplement: Figure S1 — Proliferation rate of MPs clones derived from ctrl or obese mice (p<0.05). ATP bioluminescence (RLUs) were measured in all MPs clones after 3 days of plating. (Vialight plus kit, Lonza, ME, USA) (0.15 MB TIF) [file pone.0004444.s001.tif]

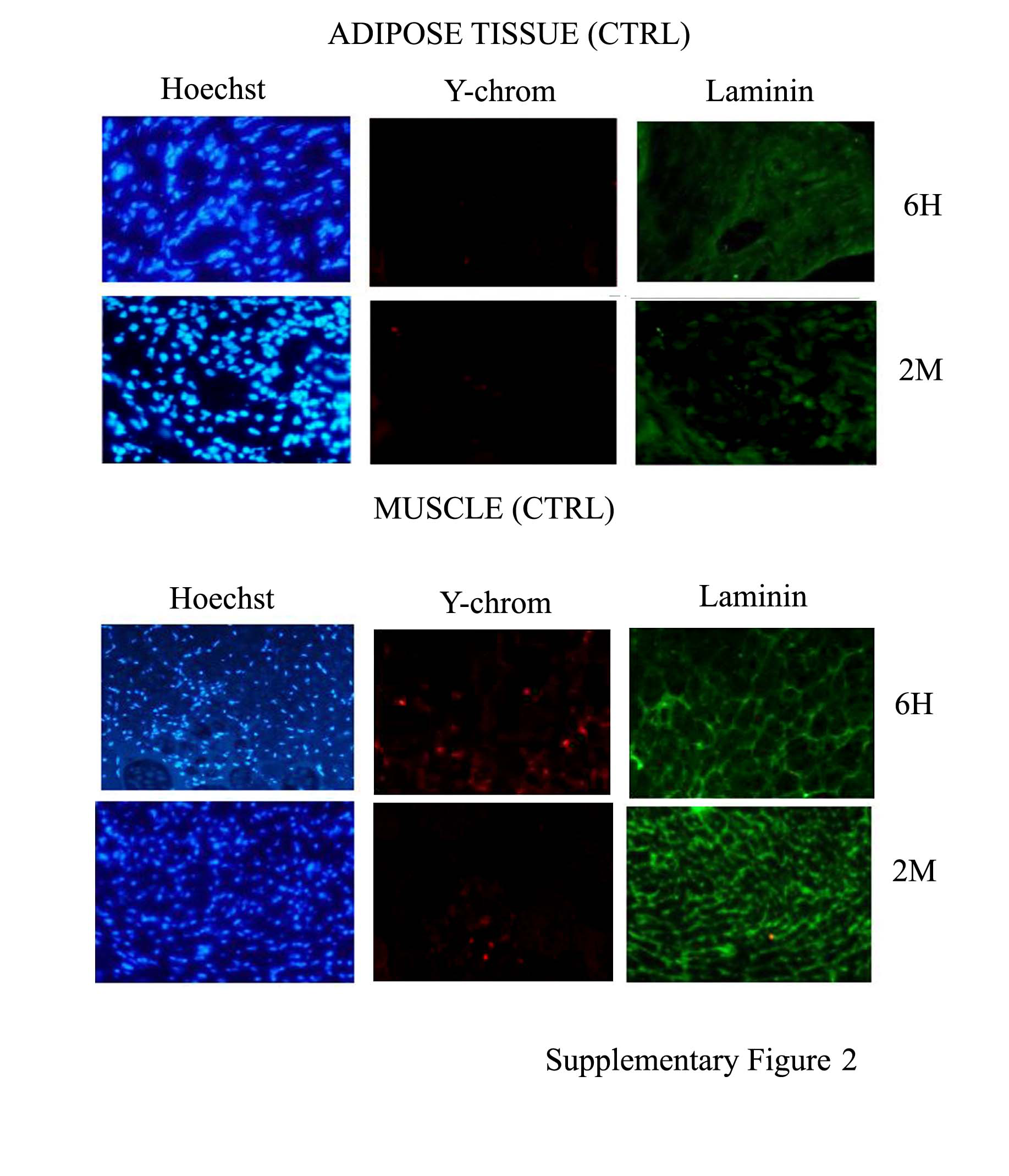

Supplement: Figure S2 — Detection of intravenously injected male MPs by immunohistology in the adipose and muscle tissue of wt mice, 6 h or 2 months after the injection. Red fluorescence stains the injected cells (Y-chromose-positive), while the green colour represents laminin staining. Hoescht dye (blue) stains all nuclei. (2.33 MB TIF) [file pone.0004444.s002.tif]

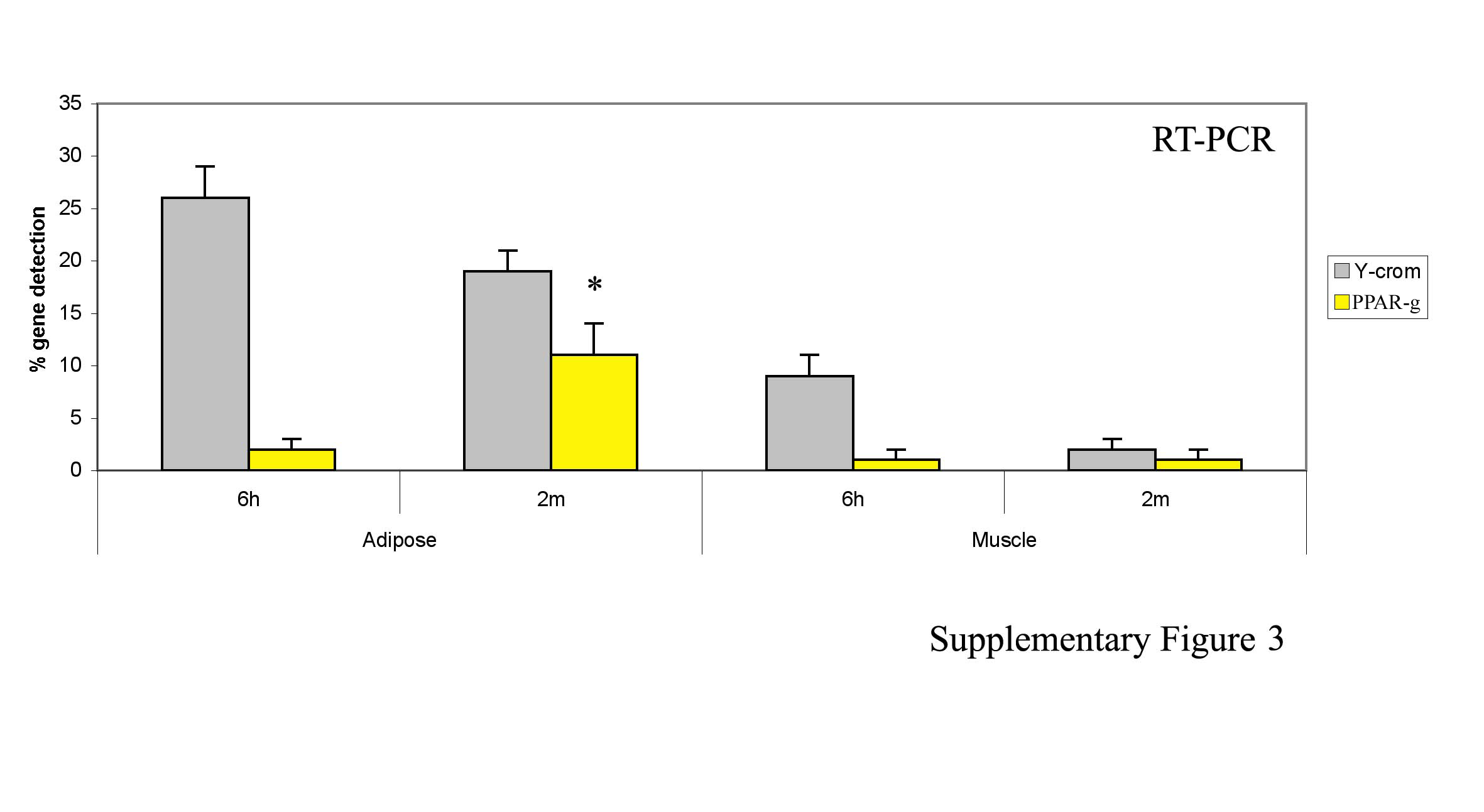

Supplement: Figure S3 — Results for epsilon chromosome (grey) and PPAR-gamma (yellow) RT-PCR, 6 h or 2 months after i.v. male MPs injection into female ob/ob mice (*p<0.05). Expression analysis was performed for the differentiation marker gene PPAR_gamma. Human glyceraldehyde-3-phosphate dehydrogenase (GAPDH) was chosen as the endogenous control. Sequences of primers for PPAR_gamma: PPAR_ Fw TCAAACACATCACCCCCCTG PPAR_ Rw TGGCAGCCCTGAAAGATGC (0.19 MB TIF) [file pone.0004444.s003.tif]

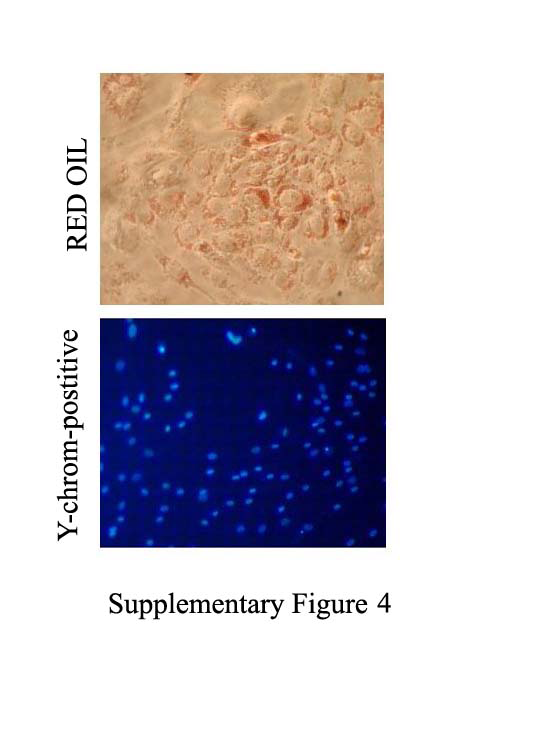

Supplement: Figure S4 — Injected male MPs were extracted from the adipose mass after two months inside the mice. Y-chromosome positive cells were isolated and their adipocyte differentiation ability was tested by Red Oil staining. (0.27 MB TIF) [file pone.0004444.s004.tif]

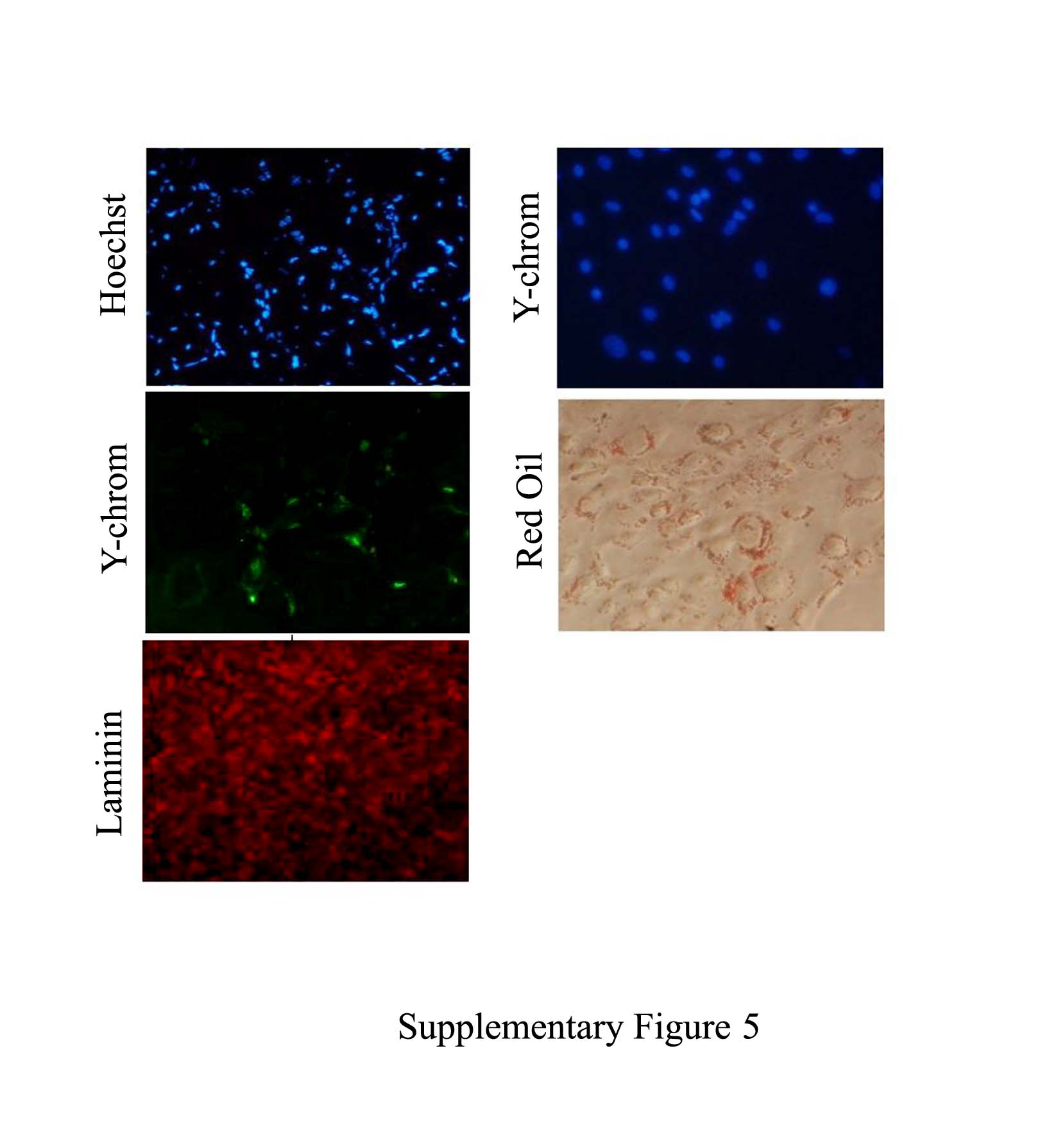

Supplement: Figure S5 — All surgeries were performed under pentobarbital sodium anaesthesia (50 mg/kg) administered intraperitoneally. For transplantation the female adipose mass pads into male SCID mice, small bilateral dorsal incisions were made, the skin and fascia were loosened using blunt tissue forceps, and the transplants were placed under the skin, which was closed with wound clips. The peritoneum and abdominal muscles were sutured, and the skin was closed with wound clips. After three weeks, fat pads were removed, with care taken to preserve the blood vessels supplying. Transplants were processed by immunocytochemistry to detect Y-chromosome positive cells. Fat pads were also processed for the isolation of the Y-chromosome positive cells and their differentiation properties analyzed by Red Oil staining. (1.12 MB TIF) [file pone.0004444.s005.tif]

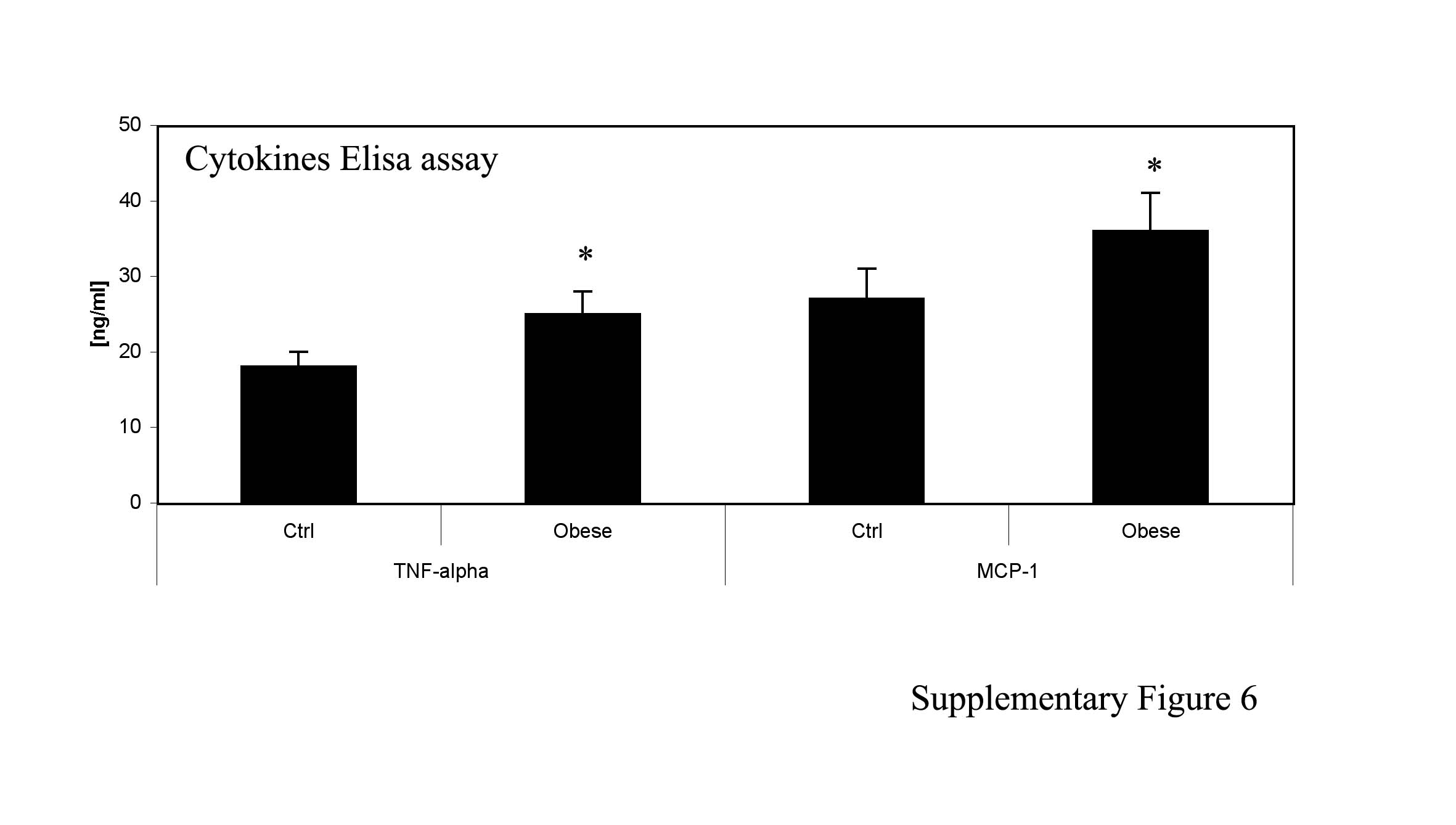

Supplement: Figure S6 — Cytokines were measured by Elisa detection kit (eBioscience). Cytokines expression is shown as ng/ml. Values are significative (*p<0.05). (0.17 MB TIF) [file pone.0004444.s006.tif]
